# *Chlamydia pneumoniae* Infection Exacerbates Atherosclerosis in ApoB100only/LDLR^−/−^ Mouse Strain

**DOI:** 10.1155/2018/8325915

**Published:** 2018-03-25

**Authors:** Ildikó Lantos, Valéria Endrész, Dezső Péter Virok, Andrea Szabó, Xinjie Lu, Tímea Mosolygó, Katalin Burián

**Affiliations:** ^1^Department of Medical Microbiology and Immunobiology, University of Szeged, Dóm Tér 10, Szeged 6720, Hungary; ^2^Institute of Surgical Research, University of Szeged, Szőkefalvi-Nagy Béla U. 6, Szeged 6720, Hungary; ^3^The Mary and Garry Weston Molecular Immunology Laboratory, Thrombosis Research Institute, Emmanuel Kaye Building, Manresa Road, London SW3 6LR, UK

## Abstract

**Aims:**

Hyperlipidaemia model animals have been used to elucidate the role of* Chlamydia pneumoniae (Cpn)* infection in atherosclerosis. The aims of this study were to investigate the proatherogenic effect of multiple* Cpn* infections in ApoB100only/LDLR^−/−^ mice which based on lipid profile can be regarded as the most suitable mouse model of human hypercholesterolemia and to compare the lesion development to that in a major atherosclerosis model ApoE^−/−^ mice.

**Methods and Results:**

Aorta samples of ApoB100only/LDLR^−/−^ mice infected three times with* Cpn* were subjected to morphometric analyses. Morphometric evaluation disclosed that* Cpn* infections exacerbated atherosclerosis development in the aortic root and descending aorta of the mice fed with normal diet. Viable Cpn was detected in the ascending aorta by RT-PCR. Chlamydial 16SrRNA expression showed the presence of viable* Cpn* in the aorta of infected animals. A similar rate of acceleration of atherosclerosis was observed when the infection protocol was applied in ApoB100only/LDLR^−/−^ and in ApoE^−/−^ mice.

**Conclusion:**

Similar to ApoE^−/−^ mice, ApoB100only/LDLR^−/−^ mice with more human-relevant serum lipoprotein composition develop increased atherosclerosis after* Cpn* infections; thus this mouse strain can be used as a model of infection-related atherosclerosis enhancement and can provide further evidence for the proatherogenic influence of* Cpn* in mice.

## 1. Introduction

Atherosclerosis is one of the most frequent causes of death in the world [1]. There are several well-known atherosclerosis risk factors, such as diabetes mellitus, smoking, hypertension, hyperlipidaemia, hypercholesterolemia, and abdominal obesity [2, 3]. The knowledge about the contributory mechanisms is incomplete [4]. Atherosclerosis begins in the early childhood [5] and has been characterized as a chronic inflammatory disease in which both innate and adaptive immune responses play a role [6]. The damage to the endothelium of the arteries is the primary cause of initiation of the development of atherosclerosis. High plasma low-density lipoprotein (LDL) cholesterol concentrations, especially oxidized LDL, contribute to the formation of the atherosclerotic lesions [7, 8].

Several infectious agents have been associated with the risk of atherosclerosis [9–12]. The infections and the accompanying inflammatory response damage the endothelial cells and also stimulate monocytes to secrete proinflammatory cytokines. Numerous studies have demonstrated an association between* Chlamydia pneumoniae (Cpn)* infection and atherosclerosis [12–14].* Cpn* infection is ubiquitous, with 50% of individuals being seropositive by 20 years of age and approximately 80% in the elderly [13, 15]. Chronic-persistent infections and reinfections are frequent which may contribute through the induced inflammation to atherosclerosis [16]. The prevalence of antibodies ranges from 60 to 80% among patients with cardiovascular diseases [17, 18]. Animal models that help to clarify the pathogenic steps and causalities in atherosclerosis play an important role in the current search for new therapeutics beyond the lipid-lowering drugs. Normal mice do not develop atherosclerosis and it requires long-term feeding of a high-fat diet to induce atherogenesis. However, there are well-established genetically modified inbred mouse lines that allow the investigation of atherosclerosis development in mouse models. The most frequently used were ApoE-deficient (ApoE^−/−^), LDL receptor-deficient (LDLR^−/−^), and human apoB100 transgenic mice which display marked atherogenesis throughout their arterial tree especially when fed with atherogenic diet [19, 20]. In ApoE^−/−^ mice, atherosclerosis develops spontaneously. However, the lipid profile in these mice is distinct from that seen in most humans with atherosclerosis; that is, apolipoprotein (apo) B48-containing LDL plasma level is high instead of apoB100 containing LDL level as in the case of humans with hypercholesterolemia [21, 22]. The mouse strain ApoB100only (ApoB^100/100^)/LDLR^−/−^ carries an* apoB* gene with a mutation preventing the expression of apoB48, the truncated form of apoB, similar to humans where no apoB editing takes place in the liver [21, 23]. LDLR deficiency prevents the uptake of apoB100 containing LDL in tissues resulting in high plasma levels of apoB100-containing cholesterol-rich LDL. The creating authors of this mouse strain described these mice as an authentic model of human familial hypercholesterolemia [21].

In mice, intranasal* Cpn* infection causes lower respiratory tract disease similar to that seen in humans with* Cpn* infection. The infection is followed by dissemination of bacteria [24] and the chlamydial DNA can be detected in the circulation of mice [25]. Most frequently, acceleration of atherosclerosis was studied after repeated* Cpn* infections which simulated chronic* Cpn* infection of humans in association with diet-induced hyperlipidaemia in C57Bl/6J [26], LDLR^−/−^ [27], or ApoE^−/−^ [28] mice and mostly found accelerated lesion development. ApoB100only/LDLR^−/−^ mice have not been used as a model for the investigation of the effect of* Cpn* infection on the atherosclerosis progression.

Our aim was to examine how repeated* Cpn* infection influences the atherosclerotic lesion development in this, based on lipid profile, most suitable mouse model of human hypercholesterolemia and atherosclerosis. We compared the extent of atherosclerosis in this model with that seen in the most frequently utilized ApoE^−/−^ mouse strain.

## 2. Methods

### 2.1. *Chlamydia pneumoniae*

*Chlamydia pneumoniae*, CWL029 strain from American Types Culture Collection (ATCC), was used.* Cpn* was propagated in HEp-2 cells (ATCC), as described earlier [29]. The partially purified and concentrated elementary bodies (EBs) were aliquoted in sucrose-phosphate-glutamic acid buffer (SPG) and stored at −80°C until use. Chlamydia was quantitated by indirect immunofluorescent method applying anti-chlamydia lipopolysaccharide monoclonal antibody (AbD Serotec, Oxford, United Kingdom) and fluorescein isothiocyanate- (FITC-) labelled anti-mouse IgG (Sigma-Aldrich, St. Louis, MO) as described earlier [30]. The number of infectious bacteria in the* Cpn* stock used for inoculation of mice was expressed as inclusion-forming units (IFU)/ml.

### 2.2. Mouse Strains

ApoB100only/LDL^−/−^ (B6.129S-Ldlr^tm1Her^Apob^tm2Sgy^/J) mice at 8-9 or 14-15 weeks of age and ApoE-deficient (ApoE^−/−^, B6.129P2-Apoe^tm1Unc^/J) mice at 14-15 weeks of age were involved in our studies. B6.129S-Ldlr^tm1Her^Apob^tm2Sgy^/J breeding pairs purchased from the Jackson Laboratory (Bar Harbor, ME, USA) and B6.129P2-Apoe^tm1Unc/J^ breeding pairs purchased from Charles River Laboratories (Sulzfeld, Germany) were housed and bred under standard conditions. Considering that numerous atherosclerosis-related studies used female mice [31–34], furthermore it was shown that female mice of both mouse strains develop atherosclerosis, with no gender-related difference in ApoE^−/−^ mice and with less extensive lesions in female than in male ApoB100only/LDLR^−/−^ mice [21, 23]; we chose to work with female mice which were available in sufficient quantity in our breeding colony.

The mice were kept on normal rodent regular chow (VRF1, Altromin GmbH & Co. KG, Lage, Germany) or high-fat/high-cholesterol diet containing 21% milkfat and 1.25% cholesterol manufactured by Altromin GmbH & Co. KG according to Teklad custom diet formula TD.19121, [composition, g/Kg: casein high protein 195.0, DL-methionine 3.0, sucrose 341.46, corn starch 150.0, anhydrous milkfat 210.0, cholesterol 12.5, cellulose (fiber) 39.0, mineral mix, AIN-76 (170915) 35.0, calcium carbonate CaCO3 4.0, vitamin mix, Teklad (40060) 10.0, ethoxyquin (antioxidant) 0.04] for 12 weeks after the first infection. All experiments were approved by the Animal Welfare Committee of the University of Szeged and conform to the Directive 2010/63/EU of the European Parliament (Permit Number: III./2187/2015.).

### 2.3. Infection with* Cpn*

Mice were intranasally infected with* Cpn*; 2 × 10^5^ IFU of* Cpn* in 20 *μ*l PBS were administered intranasally three times with 2-week intervals under mild anaesthesia by intraperitoneal injection of 100 mg/kg sodium pentobarbital. One week after each infection and at the end of the experiment at week 12, plasma samples with heparinised capillaries (Natelson blood collecting tubes, Fisher Scientific, Pittsburg, PA, USA) were harvested from the retroorbital plexus under anaesthesia as described above. For RNA detection in the aorta tissue additional groups of mice were infected once and sacrificed 1 and 4 weeks after single infection and 5 weeks after the third infection. The control animals were left uninfected.

### 2.4. Mouse Tissue Preparation and Quantification of Atherosclerosis

Twelve weeks after the first infection with* Cpn* the mice were sacrificed. In deep pentobarbital sodium anaesthesia (i.p. injection of 400 mg/kg pentobarbital sodium) hearts and aortas were perfusion-fixed using 10% buffered formalin administered through the left ventricle. The adequacy of anaesthesia was assessed by pedal withdrawal reflex in hind limbs; mice displaying no locomotor activity were processed. After formalin-perfusion, the upper part of the heart and the descending aorta were dissected. Aortic sinus samples were also collected for RNA extraction from mice anaesthetized as described above. The basis of the heart was separated from the aorta which was dissected until the iliac bifurcation. The upper part of the hearts was embedded in paraffin and sectioned for morphometric analyses according to the method described by Paigen et al. [35]. From the end of the aortic sinus 10 *μ*m sections were prepared until the point where the valve cusps disappeared and stained with hematoxylin eosin. Images of aortic root sections (8 sections/mouse, every third sections) were acquired with a light microscope (Leitz Optical microscope) and Olympus C-7070 digital compact camera. Percentage length of the plaque-covered perimeter of aorta lumen and percentage of aorta lumen area occupied by plaque were analysed with JMicroVision software. The aortic arch and the adjoining descending aorta were cleared from adjacent tissue and were opened longitudinally. The aortas were laid flat on black plastic surface and pictures of the longitudinally opened vessels were taken applying the same illumination, magnification, and focal distance using a CMOS camera (DCM 510; pixel size: 2.2 *μ*m × 2.2 *μ*m, 2592 × 1944 pixels; 5 Mpixel; Hangzhou Scopetek Opto-Electric Co., Ltd., Hangzhou, Zheijang, China) and the ScopePhoto software (Hangzhou Scopetek Opto-Electric Co., Ltd.) attached to a stereomicroscope (Alpha STO 44, Elektro-Optika Kft., Érd, Hungary). The digital image of the luminal surface was evaluated for the extent of atherosclerosis by tracing and measuring the plaque area and the total luminal surface using JMicroVision software. The percentage of the luminal area covered by plaque was calculated for each aorta sample.

### 2.5. RNA Extraction and Quantitative Real Time-PCR

Aorta sinus with aortic arch for RNA extraction was collected from mice (in deep pentobarbital sodium anaesthesia as described in the previous paragraph) at the indicated time points after* Cpn* inoculation for one time or three times and pools of 3 samples at each time points were snap-frozen in liquid nitrogen. Aorta samples from noninfected mice were also dissected parallel with the samples collected one week after one infection.

Total RNA was isolated from the pooled aorta samples with RNA extraction kit (Nucleospin RNA XS kit, Macherey-Nagel GmbH, Düren, Germany). Concentration and purity (OD260/280) of RNA was determined by spectrophotometry. The extracted RNA was treated with DNaseI (Sigma, St. Louis, MO, USA). Complementary DNA (cDNA) was synthetized from 1 *μ*g DNase-treated RNA with qScript cDNA Supermix synthesis kit (Quanta Biosciences, Gaithersburg, MD, USA). RNA and cDNA were stored at −80°C until use.

By using cDNA as template qRT-PCR was performed with PerfeCTa SYBR Green Supermix (Quanta) in CFX96 Real Time C1000 Thermal Cycler (BioRad, Hercules, CA, USA). All experiments involved control reactions containing distilled water as template.* Chlamydia* 16SrRNA and mouse *β*-actin sequences were amplified. The sequences of primers used for RT-PCR were the following:* Cpn* 16S rRNA: 5′-GGCGAAGGCGCTTTTCTAA-3′, 5′-CCAGGGTATCTAATCCTGTTTGCT-3′ [36]; mouse *β*-actin: 5′-TGGAATCCTGTGGCATCCATGAAA-3′, 5′-TAAAACGCAGCTCAGTAACAGTCCG-3′ [37].

A BLAST search was performed to check the specificity of the product target sequence of the primer sets. The primers were synthesized by Integrated DNA Technologies Inc. (Montreal, Quebec, Canada). The PCR cycles consisted of a 3 min denaturation at 95°C followed by 55 cycles each of 10 s of denaturation at 94°C, 10 s of annealing at 60°C and 10 s of extension at 72°C. The specificity of amplification was confirmed by carrying out a melting curve analysis. The sensitivity of amplification was controlled using standard as described earlier [38]. Amplicon standard was generated by amplifying* Cpn* cDNA with 16S rRNA primers; amplicons were purified with the PCR Clean-Up Kit (GeneElute PCR Clean-Up Kit, Sigma-Aldrich); the DNA concentration was measured with NanoDrop 1000 Spectrophotometer. The copy number was calculated using the following formula: copy number/*μ*l = [6.022 × 1023 (molecules/mole) × DNA concentration (g/*μ*l)]/(number of bases pairs × 660 daltons); and standard curve were generated from 10-fold serial dilutions of the amplicon (from 1000,000 to 1 copies). qPCR analysis of the dilution series showed that the sensitivity threshold of our method was ten 16SrRNA copies.

### 2.6. ELISA for Detection of* Cpn*-Specific Antibodies

Plasma samples of mice collected one week after each infection and at the end of the experiment were tested in duplicate for* Cpn*-specific IgG, IgM, and IgA by an in-house developed ELISA test as described earlier [25], where NP-40 treated partially purified* Cpn* and similarly treated Hep-2 mock preparation were used as antigens. Briefly, NUNC Maxisorp ELISA plates were coated with* Cpn* and mock antigen (0.625 *μ*g protein in 50 *μ*l PBS/well), respectively, overnight at 4°C. Blocking was done with 1% skim milk in PBS with 0.05% TWEEN 20 for 1 h. The serum samples were diluted in 0.4% skim milk in PBS with 0.05% TWEEN 20.

Mouse IgG, IgM, and IgA were detected with HRP-anti-mouse IgG (Jackson ImmunoResearch Laboratories West Grove, PA, USA), *α*-mouse IgA-HRP (Sigma), and anti-mouse IgM *μ*-chain (ab97260, Abcam, Cambridge, UK) secondary antibodies, respectively. Optical densities (OD-s) detected on* Cpn* antigen were corrected with OD values measured on the control antigen.* Cpn*-specific IgG antibody titres were determined by testing serial 2-fold dilutions of the serum samples on* Cpn* and control ELISA antigen, and reciprocal of the dilution producing OD ≥ 0.1 after correction with OD values measured on control antigen was regarded as titre. Geometric mean of titres of individual serum samples was calculated. For determination of* Cpn*-specific IgM and IgA levels serum samples were tested at 1 : 50 dilution and determined as the measured and corrected OD values.

### 2.7. Serum Lipoprotein Analysis

Levels of total cholesterol, triglycerides, high-density lipoprotein (HDL), and LDL-cholesterol were determined in plasma samples of mice through a service from the Department of Laboratory Medicine, University of Szeged, Hungary.

### 2.8. Statistical Analysis

Data are expressed as mean ± SD. Independent-samples *t*-test was used with SigmaPlot for Windows Version 11.0 software. A *P* value of less than 0.05 was considered to indicate statistically significant difference.

## 3. Results

### 3.1. Infection of ApoB100only/LDLR^−/−^ Mice with* Cpn*

Mice infected 3 times with* Cpn* showed mild symptoms of a disease as ruffled fur and moderate food consumption, especially during the first week after the first infection. At the time of the first infection half of the mice were given a high-fat/high-cholesterol diet the other half were kept on normal rodent chow. Noninfected mice were kept under similar conditions. All infected mice produced* Cpn*-specific antibodies which were not seen in the noninfected mice. Normal and high-fat/high-cholesterol diet-fed mice produced similar level of* Cpn*-specific IgG antibodies (OD 0.36–0.4 at dilution 1 : 100).

### 3.2. Repeated* Cpn* Infection Aggravates Atherosclerosis Development in the Aorta Sinus and in the Descending Aorta of ApoB100only/LDLR^−/−^ Mice Kept on Normal Diet

**Figure 1:**
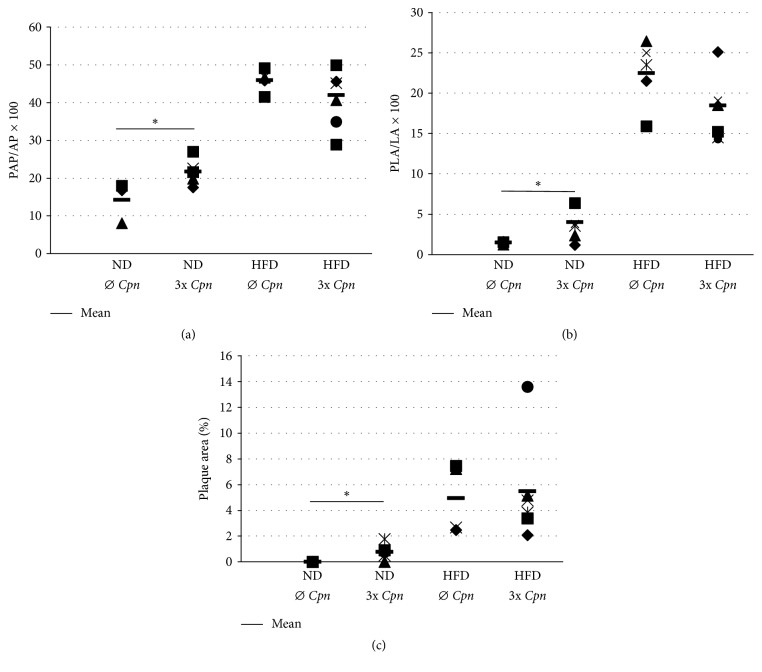
Morphometric analyses of atherosclerotic plaques in ApoB100only/LDLR^−/−^ mice. (a) Measurements for the length of the aortic luminal surface (perimeter) covered by plaque (PAP/AP × 100); (b) percentage of aortic lumen area occupied by plaques (PLA/LA × 100) in 8 cross sections of aortic root from each ApoB100only/LDLR^−/−^ mouse in groups kept on normal diet without* Cpn* infection (number of mice (*N*) = 6) or infected with* Cpn* 3 times (*N* = 8) or on high-fat/high-cholesterol diet for 12 weeks without* Cpn* infection (*N* = 6) or infected with* Cpn* 3 times (*N* = 8). Average percentage values from individual mice and mean percentages in groups are shown. PAP: plaque-covered aorta perimeter; AP: aorta perimeter; PLA: plaque-occupied lumen area; LA: aorta lumen area. (c) Plaque size was measured in the longitudinally opened descending aorta of each ApoB100only/LDLR^−/−^ mouse in groups kept on normal or high-fat/high-cholesterol diet for 12 weeks without* Cpn* infection or infected with* Cpn* 3 times. The percentage of total aorta area covered by plaques was calculated. Data show the results of one of two independent experiments. For comparison of groups independent-samples *t*-test was used, ^*∗*^*P* < 0.05. ND: normal diet; HFD: high-fat/high-cholesterol diet; 3x: three intranasal* Cpn* infections.

The mice received the first* Cpn* infection at the age of 8-9 weeks; uninfected mice with same age served as controls. Fed with an atherogenic diet very similar pathology was observed in the aorta sinus of the mice that received three chlamydia infections and in those remaining uninfected. The quantitative evaluation did not disclose significant difference in the length of the plaque-covered perimeter of the lumen (Figure 1(a)) nor in the size of the plaque-occupied area in the aorta lumen (Figure 1(b)). The aorta sections of the normal diet-fed noninfected animals demonstrated very early-stage and small extent of atherosclerosis with a single layer of foam cells. However, in the aorta sections of repeatedly* Cpn*-infected mice, we observed a significant increase in the length of the plaque-covered perimeter of the lumen (*P* < 0.05) (Figure 1(a)) and in the size of the plaque-occupied area in the aorta lumen (*P* < 0.05) (Figure 1(b)) compared with that in the noninfected counterpart in normal diet-fed group.

On the luminal surface of the longitudinally opened descending aorta well discernible plaques could be observed. Small areas corresponding to atherosclerotic alteration were seen in the case of the aorta of the noninfected animals fed with normal diet. The atherosclerosis-affected areas were measured significantly larger in the descending aorta of the infected animals. In the high-fat/high-cholesterol diet-fed animals, significantly increased plaque-covered spots were visible compared to that in the normal diet-fed mice; however, the infection did not enhance the lesion formation in this part of the aorta (Figure 1(c)).

### 3.3. Viable* Cpn* Was Detectable in the Aorta of ApoB100only/LDLR^−/−^ Mice

**Figure 2:**
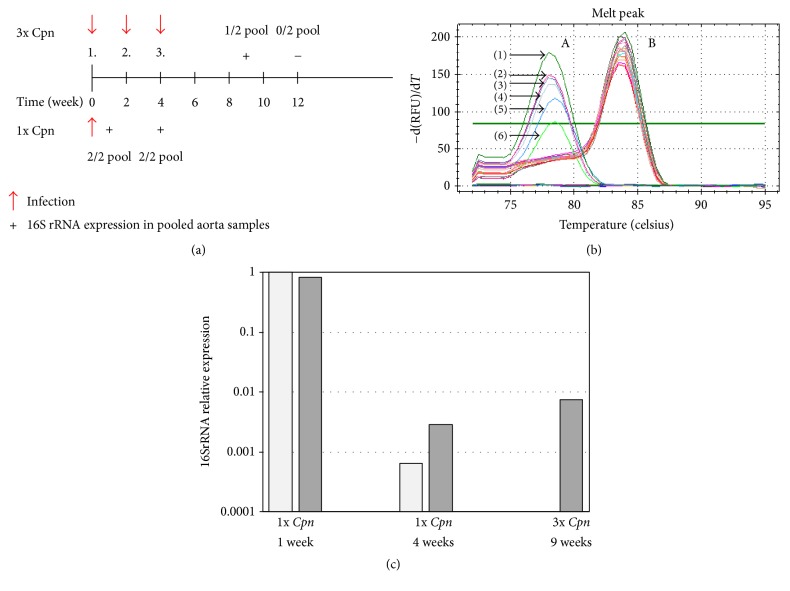
*Chlamydia* RNA in the aorta tissues. (a) Experimental design for* Cpn* infection of ApoB100only/LDLR^−/−^ mice and 16SrRNA transcript detection in pooled aorta samples (two pools of 3 aortas from 6 mice) of one time (1x* Cpn*) and 3 times (3x* Cpn*)* Cpn*-infected and noninfected mice (“+”: aorta samples tested positive; “−”: aorta samples tested negative for expression of 16SrRNA by qRT-PCR). ((b)A) Identification of the RT-PCR-amplified* Cpn* 16SrRNA cDNA in the aorta of* Cpn*-infected ApoB100only/LDLR^−/−^ mice. Melt curves show* Cpn* 16SrRNA amplicons in (1)* Cpn*-infected McCoy cells (positive control); (2), (3) in aorta samples tested 1 week after single* Cpn* infection; (4) in aorta samples 5 weeks after third* Cpn* infection, (5), (6) in aorta samples 4 weeks after a single* Cpn* infection; ((b)B) *β*-actin amplicons in all tested aorta samples and* Cpn*-infected McCoy cells. (c) Expression of* Cpn* 16SrRNA normalized to the expression level of mouse *β*-actin at 1 week after single* Cpn *infection was set as 1 and relative mouse *β*-actin-normalized* Cpn* 16SrRNA expression level in all pooled aorta samples giving positive 16SrRNA signal was calculated. The measurements were repeated two times with the same results.

Figure 2(a) shows that the persistence of the bacterium was tested early, that is, one week after the first infection and again four weeks after single infection and at 9-week and 12-week time points after three chlamydial infections by RT-PCR. An equal amount of RNA purified from pooled ascending aorta samples of mice was DNase-treated and then reverse-transcribed and mouse* β-actin* as housekeeping gene was amplified from all samples. One and four weeks after single inoculation the expression of chlamydial 16SrRNA was detectable (Figure 2(b)) with a ~1000-fold lower relative expression level at four weeks than at 1-week time point (Figure 2(c)). Five weeks after the third infection one aorta sample of 2 showed metabolically active* Cpn* in the aorta and the relative expression level was similar to that after single infection at 4 weeks (Figure 2(c)). At later time point, our test was not able to detect chlamydial gene expression.

### 3.4. Infection of ApoB100only/LDLR^−/−^ and ApoE^−/−^ Mice with* Cpn* Induces Similar Kinetics of Antibody Production

**Figure 3:**
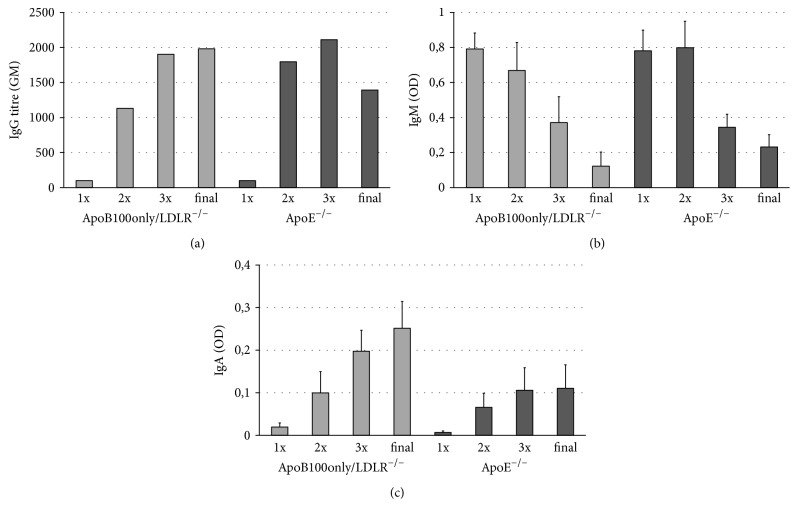
*Cpn*-specific antibodies in the mice. The serum antibody levels were measured in ApoB100only/LDLR^−/−^ and ApoE^−/−^ mice 1 week after first (1x), second (2x), third (3x)* Cpn *infection and at the end of the experiment (week 12) by an in-house ELISA test with* Cpn *antigen. (a) Geometric mean (GM) of IgG titres, reciprocal of dilutions producing OD ≥ 0.1 is shown. (b) For detection of* Cpn*-specific IgM and (c) IgA level in the sera 1 : 50 dilutions of serum samples (ApoB100only/LDLR^−/−^: *N* = 7, ApoE^−/−^ mice: *N* = 8) were tested with the ELISA test. OD values and standard deviations (SD) are shown. Data represent the results of one of two independent experiments.

Based on the above results, we aimed at performing a comparative experiment by applying our infection protocol both in ApoB100only/LDLR^−/−^ and in ApoE^−/−^ mice (14-15 weeks of age) while keeping them on a nonatherogenic diet. The humoral immune response induced by the infections was compared by measuring the titre of* Cpn*-specific IgG antibodies (Figure 3(a)) and the level of IgM (Figure 3(b)) and IgA (Figure 3(c)) antibodies one week after all three infections and at the end of the experiment. The level of antibody response did not differ significantly in the two mouse strains. The IgA level tended to be lower in ApoE^−/−^ mice (Figure 3(c)); however, the difference was not significant. Two independent experiments gave similar results.

### 3.5. The Extent of Atherosclerosis Is Similarly Increased in the Aorta of* Cpn*-Infected and Normal Diet-Fed ApoB100only/LDLR^−/−^ and ApoE^−/−^ Mice

**Figure 4:**
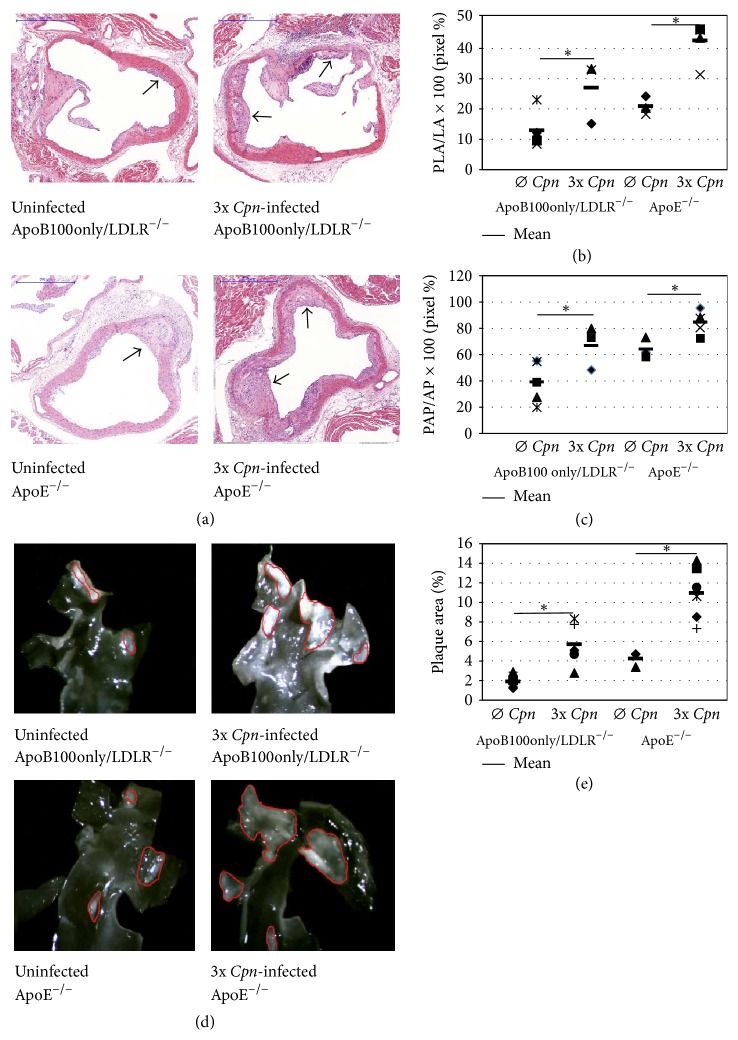
Comparative quantitative assessment of atherosclerosis in ApoB100only/LDLR^−/−^ and ApoE^−/−^ mice. Atherosclerosis development was assessed by histological and morphometric analyses in the aorta sinus and descending aorta of ApoB100only/LDLR^−/−^ and ApoE^−/−^ mice without and with 3* Cpn* infections, 12 weeks after the first infection kept on normal diet. (a) Photo of representative hematoxylin and eosin-stained cross sections of the noninfected and* Cpn*-infected ApoB100only/LDLR^−/−^ mice and noninfected and* Cpn*-infected ApoE^−/−^ mice (scale bar: 500 *μ*m). Arrows point to atherosclerotic lesions. (b) The percentage length of the aortic luminal surface (perimeter) covered by atherosclerotic plaque (PAP/AP × 100); (c) percentage of aortic lumen area occupied by atherosclerotic plaques (PLA/LA × 100) in 8 sections of aortic root from noninfected (*N* = 6),* Cpn*-infected (*N* = 7) ApoB100only/LDLR^−/−^, and noninfected (*N* = 6) and* Cpn*-infected (*N* = 8) ApoE^−/−^ mice. Average percentage values from individual mice and mean percentages in groups are shown. (d) In situ microscopic pictures of en face proximal aorta of noninfected and* Cpn*-infected ApoB100only/LDLR^−/−^ and ApoE^−/−^ mice, respectively. (e) Plaque size was measured on the length of the luminal surface of the descending aorta of 6–8 mice, and the percentage of total aorta area covered by plaques was calculated. Percentage values in individual mice and mean percentages in groups are shown. Data demonstrate the results of one of two independent experiments. For comparison of groups independent-samples *t*-test was used, ^*∗*^*P* < 0.05. PAP: plaque-covered aorta perimeter; AP: aorta perimeter; PLA: plaque-occupied lumen area; LA: aorta lumen area; 3x: three intranasal* Cpn* infections.

In normal chow-fed ApoB100only/LDLR^−/−^ mice at the age of 24-25 weeks the lesions consisted of mainly single or multiple layers of macrophage foam cells but some more advanced plaques with cholesterol crystals and necrotic core were also seen. In ApoE^−/−^ mice at same age more numerous advanced lesions with necrotic core and cholesterol cleft were found. In* Cpn*-infected ApoB100only/LDLR^−/−^ mice larger advanced plaques and in infected ApoE^−/−^ mice more plaques with necrotic core and accumulated cholesterol crystals appeared. Representative sections from the experimental groups are shown in Figure 4(a).

The measurements proved that in ApoE^−/−^ mice the atherosclerosis was more pronounced than in ApoB100only/LDLR^−/−^ mice. The difference was seen in respect of the size of plaque-occupied lumen area in the aortic sinus (Figure 4(b)) and the length of the plaque-covered aorta surface in the lumen (Figure 4(c)). Lesions in the descending aorta also were larger in ApoE^−/−^ mice than in ApoB100only/LDLR^−/−^ mice (Figures 4(d) and 4(e)). When the effect of* Cpn* infection was analysed, significant enhancement in the measured values was found. The plaque-covered perimeter of the lumen in the aorta sinus sections (Figure 4(b)), the plaque-occupied lumen area (Figure 4(c)), and the plaque size in the descending aorta (Figure 4(e)) increased 2.08-fold (*P* = 0.035), 1.7-fold (*P* = 0.004), and 2.5-fold (*P* = 0.001), respectively, in ApoB100only/LDLR^−/−^ and 2.04-fold (*P* = 0.019), 1.32-fold (*P* = 0.026), and 2.56-fold (*P* = 0.002), respectively, in ApoE^−/−^ mice.

### 3.6. Plasma Lipid Levels in ApoB100only/LDLR^−/−^ and ApoE^−/−^ Mice

**Figure 5:**
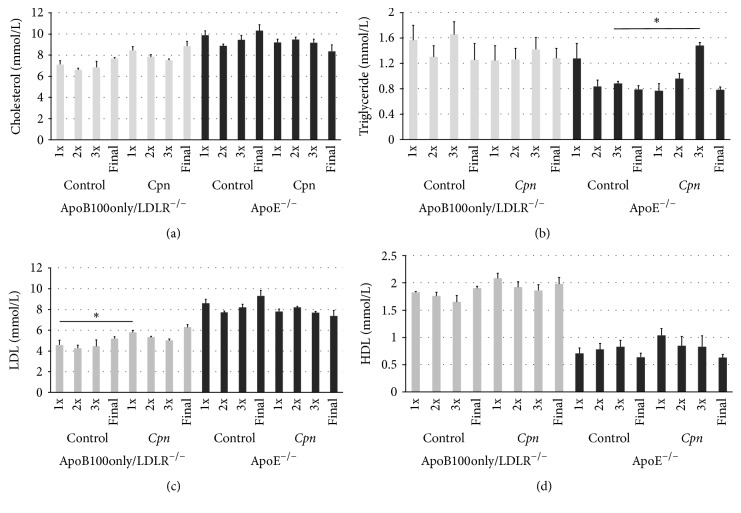
Plasma lipid levels in the mice. Plasma samples of ApoB100only/LDLR^−/−^ and ApoE^−/−^ mice taken at 1 week after the first (1x), second (2x), and third (3x)* Cpn* infection and at the end of the experiment (week 12) and from the noninfected control mice at the same time points were tested for concentration of (a) total cholesterol, (b) triglyceride, (c) LDL, and (d) HDL content. Lipid concentrations are expressed as mmol/L; mean of values measured in individual mouse sera (ApoB100only/LDLR^−/−^ noninfected (*N* = 6),* Cpn*-infected (*N* = 7), and ApoE^−/−^ mice noninfected (*N* = 6),* Cpn*-infected (*N* = 8)), and standard deviations (SD) are shown; independent-samples *t*-test was used, ^*∗*^*P* = 0.01. The figure demonstrates the results of one of two independent experiments.

Plasma samples of mice were tested for lipid levels. At each tested time point irrespective of the infection status ApoE^−/−^ mice carried a higher level of total cholesterol than the ApoB100only/LDLR^−/−^ mice (Figure 5(a)). In the plasma of uninfected ApoB100only/LDLR^−/−^ mice the triglyceride concentration was elevated compared to that in ApoE^−/−^ mice (Figure 5(b)). Infection one or two times did not cause increase in triglyceride level, but the third* Cpn* inoculation resulted in a significant elevation (*P* = 0.001) in ApoE^−/−^ mice. In ApoB100only/LDLR^−/−^ mice no infection-related change in triglyceride level was obvious (Figure 5(b)). Plasma concentration of LDL was higher in ApoE^−/−^ mice than in ApoB100only/LDLR^−/−^ mice (Figure 5(c)). Infection-related significant increase in LDL level was associated with the first infection in ApoB100only/LDLR^−/−^ mice only (*P* = 0.04). HDL plasma concentration was generally higher in ApoB100only/LDLR^−/−^ mice than in ApoE^−/−^ mice and in these mice the concentration decreased by the end of the observation period but remained high in ApoB100only/LDLR^−/−^ mice (Figure 5(d)). No infection-associated change in HDL level was detected.

## 4. Discussion

ApoE^−/−^ mice are widely used as animal models of atherosclerosis; however, the lipoprotein metabolism of this mouse strain is different from that in humans with hypercholesterolemia. ApoE^−/−^ mice accumulate in their plasma large quantities of ApoB48 containing lipoprotein of the very low-density lipoprotein (VLDL) class while humans with atherosclerosis almost always have high level of cholesterol-rich LDL containing ApoB100 [23, 31]. Many publications investigating the relation of* Cpn *with atherosclerosis have used this model to disclose the nature of the association between infection with this pathogen and initiation and/or acceleration of atherosclerosis [28, 39–42]. It has been suggested that* Cpn* infection exacerbates atherosclerosis in conjunction with hyperlipidaemia; however, ApoE deficiency might influence the immune response to this pathogen and provides increased resistance to vascular infection [41]. We aimed at examining the influence of repeated* Cpn* infection on the formation of atherosclerotic plaques in ApoB100only/LDLR^−/−^ mouse strain another model for lipoprotein abnormalities which can be regarded as the most faithful model of human familial hypercholesterolemia [21].

ApoB100only/LDLR^−/−^ mouse strain was created by genetic modification, so that majority of their plasma cholesterol is in the LDL class with ApoB100 and develops atherosclerosis on low-fat, chow diet. First, we wanted to establish that ApoB100only/LDLR^−/−^ mice can serve as a model for investigating the role of* Cpn* in atherosclerosis. Therefore groups of mice were fed with a normal or high-fat/high-cholesterol diet and were repeatedly infected with* Cpn* or left uninfected, and development of atherosclerosis was followed. As our experiments showed, in ApoB100only/LDLR^−/−^ mice which were fed with normal diet repeated three* Cpn* infections resulted in an enhanced atherosclerosis development in the aortic sinus and the descending aorta. High-fat/high-cholesterol diet-induced enhanced atherosclerosis was not exacerbated by* Cpn* infections. Thus all further experiments were done with mice kept on normal diet. As it is said that* Cpn* acts in cooperation with hyperlipidaemia it seems that the effect of hyperlipidaemia in ApoB100only/LDLR^−/−^ mice can be aggravated by* Cpn* infection but* Cpn* does not exacerbate atherosclerosis further in the presence of high-fat/high-cholesterol diet [43–45]. Nevertheless, the bacterium influenced the course of atherosclerosis development indicating that ApoB100only/LDLR^−/−^ mice are suitable for further research. Our results are consistent with findings of Moazed et al. who described atherosclerosis-accelerating effect of* Cpn* infection in ApoE^−/−^ mice eating regular chow diet [44]. However, atherosclerosis was also exacerbated in ApoE^−/−^ mice kept on high-fat diet by single or 3 repeated* Cpn* infections [39, 46].

We hypothesized that, based on the genetic difference between ApoB100only/LDLR^−/−^ and ApoE^−/−^ mice, features that characterize* Cpn* infection or atherosclerosis may also differ, and therefore we compared the effects of the bacterium in these mouse strains. In our infection model the successful infection was demonstrated by detecting* Cpn*-specific IgG, IgA, and IgM antibodies in the mice throughout the course of the experiment and at the time of sacrifice. When IgG antibody level was compared in ApoB100only/LDLR^−/−^ and ApoE^−/−^ mice, no significant difference was observed. Nazzal et al. [41] reported that ApoE^−/−^ mice produced more* Cpn*-specific antibodies than wild-type mice which was attributed to ApoE deficiency. Our results do not support this suggestion considering that in ApoB100only/LDLR^−/−^ mice repeated* Cpn* infection led to high level of antibody production without the contribution of ApoE deficiency.

Not only was antibody response the sign of infection, but we were able to detect metabolically active* Cpn* in the aorta samples for nine weeks after the first infection. Previous studies demonstrated the presence of* Cpn* in the aorta of repeatedly infected ApoE^−/−^ mice by isolating the bacterium early two weeks after infection. Furthermore, bacterial DNA was amplified by PCR at later time points [47]. As reviewed by Campbell and Rosenfeld [48] several lines of evidence point to the ability of* Cpn* to establish persistent infection* in vivo*. Our results provide additional information about persisting chlamydia as 16SrRNA gene transcripts suggest metabolically active bacteria not only persisting DNA or antigen late after repeated infections. Long-term presence of viable chlamydia in the aorta tissues of some infected mice might contribute to the atherogenic effect of the infection.

In female ApoE^−/−^ mice we have disclosed more advanced atherosclerosis than in ApoB100only/LDLR^−/−^ mice at the same age at the end of the 12-week observation period. Less pronounced difference was disclosed by Powell-Braxton et al. [21]. Nevertheless, in ApoE^−/−^ mice, similar to ApoB100only/LDLR^−/−^ mice, the infection resulted in an enhanced lesion formation without the need of feeding the mice with high-fat diet confirming the results with ApoE^−/−^ mice of Moazed et al. [44].

It has been described that* Cpn* can cause hepatic fatty acid imbalance [49] dysregulation of lipid metabolic genes in the liver [50] and altered macrophage cholesterol homeostasis [51]; however, most of the studies in mice did not detect major changes in plasma lipid profile after* Cpn* infection [34, 39]. Increase in triglyceride concentration after repeated infection of ApoE^−/−^ mice similarly to our results has been noted by Rothstein et al. [33]. The reason of increase in triglyceride level in chronic infection induced by multiple inoculations in ApoE^−/−^ mice may be the absence of ApoE. ApoE is known to provide protection against the inflammation induced by bacterial lipopolysaccharide [52] and the inflammation related change in lipid metabolism [53]. Early increase of LDL level in ApoB100only/LDLR^−/−^ mice after primary infection may also be due to the primary infection caused inflammation [54]. Our findings are concordant with results of a study by Kontula et al. [55] suggesting a significant association between chronic infection with* Cpn* and increased risk of coronary heart disease in patients with familial hypercholesterolemia.

## 5. Conclusion

In the field of infection-related exacerbation of atherosclerosis, the ApoB100only/LDLR^−/−^ mouse strain has not been utilized. According to our results the infection is followed by long-lasting vascular infection in this mouse strain contributing to the potential direct effect of the infection in the vessel wall. As ApoE deficiency may alter the immune response against* Cpn* infection compared to wild-type mice, ApoB100only/LDLR^−/−^ mice might provide additional information regarding the immune mechanisms participating in the* Cpn* induced acceleration of atherosclerosis. The results of our experiments further support the proatherogenic role of* Cpn* infection in a model of human familial hypercholesterolemia. As repeated* Cpn* inoculation is able to aggravate lesion development in association with the lipoprotein abnormalities without feeding high-fat/high-cholesterol diet to these mice, we suggest using this mouse strain as an alternative model to investigate the role of infection in atherosclerosis development.
